# Clustering of cardiovascular behavioral risk factors and blood pressure among people diagnosed with hypertension: a nationally representative survey in China

**DOI:** 10.1038/srep27627

**Published:** 2016-06-09

**Authors:** Yichong Li, Xiaoqi Feng, Mei Zhang, Maigeng Zhou, Ning Wang, Limin Wang

**Affiliations:** 1 National Center for Chronic and Non-communicable Disease Control and Prevention, Chinese Center for Disease Control and Prevention, Beijing, China; 2 School of Health and Society, University of Wollongong, Australia; 3 National Center for AIDS/STD Control and Prevention, Chinese Center for Disease Control and Prevention, Beijing, China

## Abstract

This study aimed to examine association between the number of behavioral risk factors and blood pressure (BP) level among a nationally representative sample of Chinese people diagnosed with hypertension. A total of 31,694 respondents aged 18+ years with diagnosed hypertension were extracted from the 2013–2014 China Chronic Disease and Risk Factor Surveillance. BP of each respondent was classified into six levels according to criteria in 2007 Guidelines for the Management of Arterial Hypertension. Information for smoking, alcohol drinking, fruit and vegetables consumption, physical inactivity, and overweight and obesity were obtained. The average number of risk factors was determined by BP level to explore potential risk factor clustering. Ten generalized proportional odds models were used to examine association between clustering of behavioral risk factors and BP level. A clear gradient between the number of behavioral risk factors and BP level was observed for men and women (P < 0.05 for both genders). BP level for men and women was much likely to upgrade as number of risk factors accumulated (P < 0.01 for 10 models). Behavioral modifications may decrease BP, and combinations of two or more behavioral interventions could potentially result in even better BP management among people diagnosed with hypertension.

Although both preventable and manageable^1^,^2^,^3^,^4^,^5^, hypertension was the top leading risk factor in China in 2013, accounting for 14.2% of total disability adjusted life years and 2.5 million deaths^6^. A rapid increase in prevalence of hypertension has been repeatedly observed in several nationwide surveys^7^,^8^,^9^,^10^,^11^ and some regional studies^12^,^13^,^14^,^15^ across the last three decades. The most recent prevalence for hypertension in China reported from a nationally representative survey was 33.5%, indicating nearly 330 million Chinese residents aged 18 years and over are directly affected^16^. Little progress has made with regards to hypertension management in China, with just 5% of people living with hypertension having their blood pressure (BP) adequately controlled^8^.

Antihypertensive medication is necessary to manage BP among people living with hypertension, however, for communities where such medication is not available or affordable, pharmaceuticals cannot be exclusively relied upon. Many modifiable behavioral risk factors are known contributors to the development of hypertension, including overweight and obesity^17^,^18^, physical inactivity^19^, poor diet^20^, excessive alcohol consumption^21^, and tobacco smoking^22^. Modification of a single one of these behaviors, or several at the same time, has proven to be effective for managing hypertension^1^,^5^,^23^. Behavioral modification is therefore recommended for all people living with hypertension, regardless of their access to appropriate medication^24^. Nevertheless, the degree that clustering of these behavioral risk factors affect hypertension management among hypertensive population has not been evaluated. In this study, we examined association between the number of behavioral risk factors and BP level among a nationally representative sample of Chinese population known to be living with hypertension.

## Results

Table 1 shows the sample size and characteristics of the sample. Level 4 accounted for the largest percentage at 37.4% of the sample (11,862 out of 31,694 respondents). A total of 7113 respondents had level 5 BP and 3354 had level 6, accounting for 22.4% and 10.6% in the sample, respectively. Those with level 1, 2,or 3 BP combined accounted for 29.5% of the sample. More than one third of respondents had age between 55 and 64 years. More respondents with higher BP level were found in higher age groups. About 59.0% of the sample were women, 51.9% came from urban areas, 82.1% had an education level of junior high school or below, and 82.9% were married or cohabiting with partners. The proportion of respondents with higher socioeconomic circumstances (for both education and income) were larger in lower BP level group than were those with an upper BP level. This indicated that BP control was better among those with higher socioeconomic circumstances. The percentages of those who received medication for their raised BP increased as BP level upgraded.

The prevalence of all five behavioral risk factors among the sample is shown in Table 2. All five behavioral risk factors were common in men regardless of their BP level. The prevalence of current smoking, Harmful use of alcohol, low fruit and vegetable intake, and physical inactivity did not vary by BP level (P > 0.05 for all), whilst respondents with level 5 BP had highest prevalence of overweight and obesity (P < 0.01). With regards to female respondents, low intake of fruit and vegetable, physical inactivity, and overweight or obesity were prevalent across each BP level. The prevalence of insufficient fruit and vegetable intake, physical inactivity, overweight or obesity were significantly higher in females with poor BP management (P < 0.01 for all). Smoking and excessive alcohol consumption were less prevalent among women and similar among groups with different BP level.

**Figure 1:**
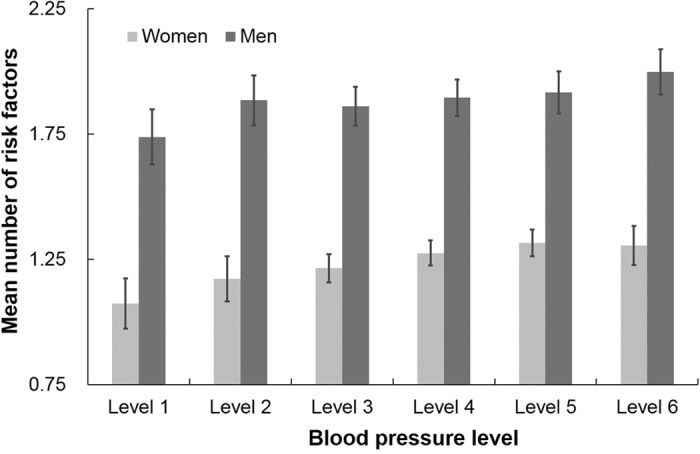
Mean number of risk factors and 95% CI among male and female hypertensive population with different blood pressure level*. *All analyses were weighted. Bars indicate 95% confidence intervals taking into account complex sample design.

We also explored the extent to which the 5 behavioral risk factors clustered within hypertensive individuals by gender (Fig. 1). Male respondents with level 1 BP had the fewest behavioral risk factors (1.74, 95% CI: 1.63–1.85), while those with level 6 BP had the most (2.00, 95% CI: 1.91~2.09). Similarly for females, those with level 1BP on average had the fewest behavioral risk factors at 1.07 (95% CI: 0.97~1.18), while those with level 5 BP had the most (1.32, 95% CI: 1.28~1.37). A clear gradient between the number of behavioral risk factors and poor management of BP was observed for men and women (P < 0.05 for both).

As generalized proportional odds model allowed varying coefficient of covariates across different cut-off point equations, table 3 shows associations between behavioral risk factors clustering on BP management for each equation by gender. After controlling for demographic characteristics and socioeconomic circumstances, both models for males and females living with hypertension indicated that BP level was much likely to upgrade as number of risk factors accumulated (P < 0.01 for all models). If the number of risk factors increased by one, on average, the likelihood of BP rising to a higher level increased by 9%~13% for males and by 12~20% for females, holding other variables constant. The effects on BP level of all covariates or potential confounding factors were presented in the online supplementary table.

## Discussion

Using data from a nationally representative survey, we examined the prevalence and clustering of five behavioral risk factors among individuals living with hypertension across different BP level, as well as associations between behavioral risk factor clustering and BP management. We found that unhealthy behaviors were rather common among the hypertensive. The clustering of behavioral risk factors was associated with higher BP level. Anti-hypertension medication and behavioral modification needs to be reinforced to improve hypertension control in China.

The high prevalence of behavioral risk factors could impede BP management among individuals living with hypertension. In our study, we found five behavioral risk factors, all of which had been previously reported in many observational studies or clinical trials to be independently associated with raised BP or risk of hypertension development^17^,^18^,^19^,^20^,^21^,^22^,^23^, were prevalent among male respondents. For example, except for patients with level 1 BP, more than half of the sample were overweight or obese, the most powerful predictor of hypertension incidence found in some studies^4^,^18^. For women, approximately 50% had low intake of fruit and vegetables; about 15% were physically inactive; and half are overweight. These results indicate huge obstacles for controlling BP among people living with hypertension in China.

Our data showed that more risk factors clustered among those respondents with poor BP management. Men with level 1 BP had only 1.74 risk factors on average, which was less than those with level 6 (2.00). Analogously, female patients with level 5 had 1.32 risk factors, nearly 25% more than those with optimal BP (1.07). This could be explained by probable cumulative effects on BP reduction from simultaneous modification of multiple risk factors. Take the PREMIER clinical trial^3^, by 6 months, the mean net reduction in systolic BP was 3.7 mm Hg in the established group (a behavioral intervention that implemented established recommendations) and 4.3 mm Hg in the established plus dietary approaches to stop hypertension (DASH) group, compared with the advice only group. Hypertension prevalence was reduced by 31.5% in the advice only group, by 55.2% in the established group, and by 68% in the established plus DASH group. Although some studies found that the cumulative effects of comprehensive interventions with several risk reduction strategies was less than expected based on interventions conducted separately^2^,^25^,^26^, adoption of all behavioral modifications was recommended as an indispensable component of BP management for people living with hypertension^24^,^27^

In the ordinal logistic regression, we found that less risk factors were consistently associated with better BP control, but treatment in last two weeks were not (supplementary table). A healthy behavioral and good compliance to medication could lead to a preferable control of hypertension. Besides prevalent risk factors among people living with hypertension, another likely reason for this would be poor efficacy of hypertension management and treatment. A national survey revealed that 44% of people living with hypertension were included in a management program by a primary health care provider, and only 35.3% were managed following standard guideline^28^. The lack of professional health staff and essential anti-hypertensive medication may result in the low treatment and control rate of hypertension, particularly in less affluent and remote communities in China.

Our study has some limitations. Firstly, the cross-sectional nature of the study precluded causal inference. Secondly, the present study might also suffer from recall bias of self-reported hypertension and behavioral risk factors. Thirdly, the survey did not include any questions on how these self-reported hypertension were detected. Some bias might be introduced due to diverse diagnosis criteria possibly adopted by doctors from health facilities at different level or from different regions in China. However, the Basic Public Health Services of China has been conducting various activities to promote hypertension detection and management. It also requires BP measurement at the first time when an individual aged 35+ years visits doctor at any health care facilities. The Chinese government has invested substantially in capacity building for the grass root doctors and the Guideline for Prevention and Treatment of Patients with Hypertension has been widely distributed^29^. We, therefore, believe the self-reported hypertension was valid and reliable. Fourthly, only five behavioral risk factors were included and some important dietary risk factors were not taken into account such as sodium and fat intake for data was unavailable. However, the large sample size, objective measurement of BP and the national representativeness of the sample design enhance internal validity and the generalizability of the results to the entire mainland Chinese hypertensive individuals.

In conclusion, combinations of two or more behavioral interventions may result in better BP control. On the basis of national or regional surveys in recent decades showing an alarming increase in hypertension prevalence^7^,^8^,^10^,^12^,^13^,^14^,^15^, there is an urgent public health need for improved behavioral intervention programs, including those appropriate for applying in the primary health care setting, that motivate individuals with or at risk for hypertension to adopt long-term healthier behaviors.

## Materials and Methods

### Data source

The data used in the study came from the 2013–2014 China Chronic Disease and Risk Factor Surveillance (CCDRFS). The CCDRFS is an on-going nationally representative surveillance system administered by the National Center for Chronic and Non-communicable Disease Control and Prevention, which is affiliated to the Chinese Center for Disease Control and Prevention. The 2013–2014 CCDRFS survey was approved by the ethics committee of the Chinese Center for Disease Control and Prevention and written informed consent was obtained from each respondent. Face-to-face interviews were done in the participant’s local language between August 2013 and July 2014. The 2013–2014 survey of the CCDRFS adopted multistage stratified random sampling to reach a nationally representative sample. A total of 298 survey sites (districts/counties) were randomly selected from all 31 provinces, autonomous regions and municipalities in mainland China, with stratification by high/low population size and high/low mortality rate in each province. Within each sampled survey site, 4 townships were selected using the method of probability proportional to population size. Then, 3 villages and residential areas were selected from each sampled township using the same sampling method as used in the previous stage. Subsequently a residential group including at least 50 households was chosen from each sampled village or residential blocks by simple random sampling. Households were eligible if 1 or more members aged 18+ years who had stayed in the survey site at least for 6 months in the last 12 months before the survey. Finally, an adult (18+ years) was selected in each family using Kish grid method. About 6.3% of the sampled families could not be accessed on three attempts in different 3 days and these households were replaced by others having a similar family structure. The survey finally included 176,740 respondents aged > 18 years in mainland China. A total of 31,694 individuals who were aware of their hypertension were extracted from the survey database for analysis.

### Measures

Cardiovascular behavioral risk factors and biomarkers were measured for each respondent in the 2013–2014 CCDRFs. For details of the survey questionnaires, standard operation procedures for physical measurements and laboratory tests, one could refer to previous publications^30^,^31^.

In a room with constant temperature around 25 °C, sitting blood pressure was measured by trained and qualified field workers following a standard operating procedure using an Omron digital BP device (Omron “HBP-1300, Omron Healthcare, Inc., Kyoto, Japan) provided for all survey sites. Vigorous activity, beverage containing caffeine like tea and coffee, and long-time exposure to cold weather were required to avoid in 1 hour before the blood pressure measurement. Left arm measurement without cloth was preferred but not for the individuals with left arm disorders or disability. Before taking any anti-hypertensive medicines on the survey day, all respondents had their BP measured 3 times successively with one-minute interval between each measurement. The average of the last two measures was used for analyses. Blood pressure readings were recorded in paper based questionnaire and double input in the online data collection system. Quality control was performed by national, provincial and local designated staff following a vigorous protocol.

Based on the measured BP, participants were classified into six BP level categories, following the definition in 2007 Guidelines for the Management of Arterial Hypertension^32^, i.e. level 1 (SBP < 120 mmHg and DBP < 80 mmHg); level 2 (SBP: 120–129 mmHg and/or DBP: 80–84 mmHg); level 3 (SBP: 130–139 mmHg and/or DBP: 85–89 mmHg);level 4 (SBP: 140–159 mmHg and/or DBP: 90–99 mmHg); level 5 (SBP: 160–179 mmHg and/or DBP: 100–109 mmHg); level 6 (SBP > 180 mmHg and/or DBP >110 mmHg). If SBP and DBP fell in range of different BP levels, the higher BP level was assigned to that individual. We assumed BP level was the natural outcome of any antihypertensive measures adopted by those hypertensive individuals, either by medical treatment, lifestyle modification, or no measures taken at all. Respondents whose BP were confined within level 1, 2, or 3 were considered as controlled. Awareness of hypertension status, previously diagnosed by health professionals from health care facility at township level or above, was self-reported by each participant.

Five behavioral risk factors were included to examine their prevalence among Chinese hypertensive population who were aware of the condition, and their associations with BP management. These risk factors were: current smoking status; harmful use of alcohol; insufficient intake of fruit and vegetables; physical inactivity; overweight and obesity. All risk factors were dichotomized as defined by Chinese standards for healthy alcohol consumption^33^ and by the WHO’s global monitoring framework of non-communicable diseases (NCDs)^34^ for others. Current smoking was defined as self-reported tobacco use every day or on some days at the time the survey was conducted. Individuals who claimed they did not smoke during the survey period were classified as non-smokers. Harmful use of alcohol was defined as daily consumption of pure alcohol ≥15 g for women and ≥25 g for men, according to the Dietary Guidelines for Chinese Residents^33^. The computation for pure alcohol consumption from various kinds of alcoholic beverages was described in detail elsewhere^30^,^35^. Food Frequency Questionnaires were used to assess fruit and vegetables intake. Consuming less than 400 g of fruit and vegetables per day was considered as insufficient. Global Physical Activity Questionnaire were used to evaluate physical activity of each respondent. Individuals with less than 150 minutes of moderate-intensity activity per week or equivalent were defined as insufficiently active. Actual height and weight were measured for all respondents, from which body mass index (BMI) was computed. Those with BMI ≥25 kg/m^2^ were classified as overweight or obese.

We also extracted information on demographic characteristics (age, gender and marital status), socioeconomic circumstances (educational level and annual per capita household income), place of residence (rural/urban) and self-reported treatment of hypertension in the past two weeks before the survey with antihypertensive medication prescribed by health professionals. Subcategories for these variables are shown in table 1.

### Statistical analysis

We described demographic characteristics, socioeconomic circumstances and other study variables for the subjects included according to their BP levels. Considering the non-trivial gender differences in some behavioral risk factors in China, such as smoking and drinking, all analyses below were conducted for men and women separately. Weighted prevalence and 95% confidence intervals (95% CI) of the five behavioral risk factors were examined by BP levels for each gender. Weights were calculated according to the sampling scheme and post-stratified by the 2010 census population. Complex sample design was taken into account in the CI estimation. Rao-Scott chi-square test was used to test overall differences of prevalence between BP levels. The average number and 95% CI of behavioral risk factors were also determined by BP level to explore clustering of behavioral risk factors among people living with hypertension with different BP management. As the proportional odds assumption was violated, a multiple ordinal logistic regression with generalized proportional odds was fitted to examine association between the clustering of behavioral risk factors and BP management. The generalized proportional odds model relaxed the proportional odds assumption and allowed associations with each explanatory variable to vary with the point at which the categories of the dependent variable are dichotomized^36^,^37^. In the model, ordered BP level was entered as dependent variable, and the mean number of behavioral risk factors was entered as independent variable, along with potential confounders such as age, sex, marital status, education, income, rural/urban residency and treatment of raised BP. It can be written as:


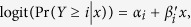


where *i* was BP level, dependent variable were dichotomized, and 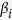 is different slopes for covariate vector *x* of every logit *i*. As there were six BP levels, five unparalleled models were constructed for each gender. All analyses were performed in SAS 9.4 (SAS Institute Inc., Cary, USA).

## Additional Information

**How to cite this article**: Li, Y. *et al*. Clustering of cardiovascular behavioral risk factors and blood pressure among people diagnosed with hypertension: a nationally representative survey in China. *Sci. Rep.* **6**, 27627; doi: 10.1038/srep27627 (2016).

## Supplementary Material

Supplementary Information

## Figures and Tables

**Table 1:** Characteristics of study population by blood pressure levels (%)*.

	Total (N)	Level 1^a^	Level 2	Level 3	Level 4	Level 5	Level 6
Total (N)	31,694	1486	2896	4983	11,862	7113	3354
Age (years)							
18–34	539	4.2	1.7	1.9	1.4	1.4	1.7
35–44	2172	8.6	8.1	6.9	6.4	6.8	6.6
45–54	7001	22	23.7	22.7	22.3	21.3	20.7
55–64	11,178	32.2	35.7	36.9	36.3	34.7	31.3
65–74	7649	23.7	21.7	23.1	24	24.7	27.1
75+	3155	9.2	9.2	8.5	9.5	11	12.6
Sex							
Men	12,994	39.0	40.4	41.8	41.5	41.1	39.2
Women	18,700	61.0	59.6	58.2	58.5	58.9	60.8
Place of residence							
Urban	16,435	57.1	58.9	57.5	53.3	47.0	40.3
Rural	15,259	42.9	41.1	42.5	46.7	53.0	59.7
Education							
Illiterate or some primary school	11,175	33.1	30.1	31.1	33.7	38.7	45.0
Primary school graduate or some junior high school	6513	18.5	18.9	19.7	20.5	21.8	21.6
Junior high school graduate or some senior high school	8338	26.4	26.6	27.9	27.4	25.0	22.4
Senior high school graduate or some college	4207	15.7	16.6	15.1	13.7	11.4	9.0
College graduate or above	1461	6.3	7.7	6.3	4.6	3.0	2.0
Marital status							
Single	334	1.1	0.8	1.1	1.0	1.1	1.2
Married or cohabiting	26,261	83.9	84.0	83.8	82.9	82.5	80.6
Separated/divorced/widowed/others	5099	14.9	15.2	15.1	16.0	16.5	18.2
Annual per capita household income^b^ (US $^c^)							
Don’t know/not sure/refused	7825	22.1	22.9	24.2	24.0	26.1	27.6
<895	6382	21.5	17.6	17.2	19.2	22.3	24.9
895–1789	4004	10.6	11.5	11.5	12.9	13.2	13.9
1790–5372	6917	21.5	21.8	22.7	22.6	21.1	19.6
>5372	6566	24.3	26.2	24.4	21.4	17.2	14.0
Medication for raised blood pressure in last two weeks							
Yes	7362	27.6	20.8	20.3	22.1	25.5	26.7
No	24,332	72.4	79.2	79.7	77.9	74.5	73.3

^*^The numbers in the columns are percentages of study sample by certain characteristics.

^a^Blood pressure level: level 1 (SBP < 120 mmHg and DBP < 80 mmHg); level 2 (SBP: 120–129 mmHg and/or DBP: 80–84 mmHg); level 3 (SBP: 130–139 mmHg and/or DBP: 85–89 mmHg); level 4 (SBP: 140–159 mmHg and/or DBP: 90–99 mmHg); level 5 (SBP: 160–179 mmHg and/or DBP: 100–109 mmHg); level 6 (SBP > 180 mmHg and/or DBP > 110 mmHg).

^b^Based on the exchange rate of 6.70 Chinese Yuan Renminbi per US dollar that was in effect on 30 September 2010.

^c^US $, United States dollar.

**Table 2:** Prevalence (%) and 95% CIs of behavioral risk factors by blood pressure levels*.

	Blood pressure levels^a^	Current smoking^b^	Harmful use of alcohol^c^	Insufficient fruit and vegetable intake^d^	Physical inactivity^e^	Overweight or obesity^f^
Male	Level 1	50.9(44.4,57.4)	14.8(9.3,20.3)	45.0(36.6,53.4)	19.3(14.0,24.7)	43.8(37.0,50.6)
	Level 2	43.7(37.3,50.2)	14.9(11.2,18.6)	46.6(38.8,54.4)	24.2(14.7,33.8)	59.0(52.7,65.3)
	Level 3	45.3(41.5,49.2)	18.3(15.3,21.3)	46.0(41.4,50.7)	19.2(16.2,22.3)	57.2(53.4,61.0)
	Level 4	45.9(43.2,48.5)	19.5(17.3,21.7)	49.3(45.6,52.9)	18.4(16.2,20.7)	56.4(53.1,59.7)
	Level 5	45.5(42.5,48.5)	20.2(17.5,23.0)	48.2(43.8,52.5)	17.5(14.9,20.1)	60.2(56.5,63.9)
	Level 6	46.1(41.4,50.9)	19.3(15.4,23.2)	52.0(47.2,56.7)	23.1(18.4,27.9)	59.2(54.1,64.4)
	P value of difference test	0.76	0.13	0.53	< 0.18	< 0.01
Female	Level 1	2.6(1.5,3.6)	1.0(0.1,1.8)	39.7(33.3,46.1)	15.7(9.2,22.2)	48.4(41.5,55.4)
	Level 2	3.5(2.1,4.9)	3.1(0.0,7.0)	47.2(42.6,51.7)	11.7(8.4,14.9)	51.7(47.4,56.1)
	Level 3	2.9(1.9,3.8)	1.3(0.5,2.1)	47.2(43.1,51.2)	14.2(11.6,16.8)	55.9(52.6,59.2)
	Level 4	3.5(2.8,4.2)	1.2(0.7,1.6)	49.5(45.8,53.2)	14.0(12.4,15.7)	59.3(56.7,61.9)
	Level 5	2.8(1.9,3.7)	1.4(0.8,2.1)	51.7(48.0,55.4)	15.9(13.5,18.2)	59.8(57.3,62.2)
	Level 6	4.7(0.0,9.5)	0.9(0.4,1.5)	52.6(47.1,58.1)	17.6(14.2,21.0)	54.7(49.9,59.5)
	P value of difference test	0.72	0.14	<0.01	<0.11	<0.01

^*^All analyses were weighted and confidence intervals (CIs) had taken into account complex sample design.

^a^Blood pressure level: level 1 (SBP < 120 mmHg and DBP < 80 mmHg); level 2 (SBP: 120–129 mmHg and/or DBP: 80–84 mmHg); level 3 (SBP: 130–139 mmHg and/or DBP: 85–89 mmHg); level 4 (SBP: 140–159 mmHg and/or DBP: 90–99 mmHg); level 5 (SBP: 160–179 mmHg and/or DBP: 100–109 mmHg); level 6 (SBP > 180 mmHg and/or DBP > 110 mmHg).

^b^Use of tobacco every day or on some days at the survey time.

^c^Consumption of ≥15 g of pure alcohol per day or women or ≥15 g per day for men.

^d^Consumption of <400 g of fruit and vegetables per day.

^e^<150 minutes of moderate activity or their metabolic equivalent per week.

^f^Body mass index ≥25 kg/m^2^.

**Table 3:** Effects of clustering of risk factors on BP levels and accumulative ORs in the generalized proportional odds models by sex*.

Cut–off points to dichotomize dependent variable	Regression coefficient of number of Risk factors	P value	Accumulative OR(95% CI)
Models for men			
≥level 6 BP^a^	0.09	0.03	1.09(1.03,1.15)
≥level 5 BP	0.08	0.02	1.08(1.04,1.12)
≥level 4 BP	0.08	0.02	1.08(1.04,1.12)
≥level 3 BP	0.10	0.02	1.10(1.05,1.16)
≥level 2 BP	0.12	0.04	1.13(1.04,1.23)
Models for women			
≥level 6 BP	0.11	0.03	1.12(1.05,1.18)
≥level 5 BP	0.11	0.02	1.12(1.08,1.16)
≥level 4 BP	0.13	0.02	1.14(1.10,1.19)
≥level 3 BP	0.18	0.03	1.20(1.14,1.27)
≥level 2 BP	0.16	0.04	1.17(1.08,1.28)

^*^Models controlled for age, marital status, income, education, rural/urban residency, medication on BP.

^a^Blood pressure level: level 1 (SBP < 120 mmHg and DBP < 80 mmHg); level 2 (SBP: 120–129 mmHg and/or DBP: 80–84 mmHg); level 3 (SBP: 130–139 mmHg and/or DBP: 85–89 mmHg); level 4 (SBP: 140–159 mmHg and/or DBP: 90–99 mmHg); level 5 (SBP: 160–179 mmHg and/or DBP: 100–109 mmHg); level 6 (SBP > 180 mmHg and/or DBP > 110 mmHg).
