# Supplementary material for: Clustering of cardiovascular behavioral risk factors and blood pressure among people diagnosed with hypertension: a nationally representative survey in China
Source: Sci Rep. 2016 Jun 9;6:27627. doi: 10.1038/srep27627 (PMC4899782; doi:10.1038/srep27627)
Supplement: Supplementary Information [file srep27627-s1.pdf]

Article title: Clustering of cardiovascular behavioral risk factors and blood pressure among people diagnosed with hypertension: a nationally representative survey in China

Author list:Yichong Li, Xiaoqi Feng, Mei Zhang, Maigeng Zhou, Ning Wang, Limin Wang

Supplementary Table. Effects of each covariates on BP levels and accumulative ORs in the generalized proportional odds models by sex.

		⩾level 6 BP [a]			⩾level 5 BP		
		Regression coefficient	OR (%95 CI)	*P*	Regression coefficient	OR (%95 CI)	*P*
Men	Education (reference: Illiterate or semi-illisteate)						
	College graduate or above	-0.87	0.42(0.30,0.59)	<0.01	-0.50	0.61(0.50,0.73)	<0.01
	Senior high school graduate or some college	-0.24	0.79(0.65,0.96)	0.02	-0.15	0.86(0.75,0.98)	0.02
	Junior high school graduate or some senior high school	-0.28	0.76(0.64,0.89)	<0.01	-0.11	0.89(0.80,0.99)	0.04
	Primary school graduate or some junior high school	-0.25	0.78(0.66,0.92)	<0.01	-0.04	0.96(0.86,1.07)	0.46
	Marital status (reference: single)						
	Married or cohabiting	-0.05	0.95(0.63,1.43)	0.81	-0.02	0.98(0.74,1.29)	0.87
	Separated/divorced/widowed/others	0.07	1.07(0.69,1.66)	0.76	0.01	1.01(0.75,1.36)	0.93
	Age (reference: 18-24 years)						
	35-44 years	0.10	1.10(0.71,1.72)	0.66	-0.00	1.00(0.75,1.32)	0.98
	45-54 years	-0.06	0.94(0.62,1.43)	0.77	0.01	1.01(0.77,1.32)	0.96
	55-64 years	-0.13	0.88(0.58,1.33)	0.55	-0.02	0.98(0.75,1.28)	0.88
	65-74 years	0.02	1.02(0.67,1.55)	0.92	0.10	1.11(0.85,1.45)	0.46
	75+ years	0.12	1.12(0.72,1.75)	0.61	0.19	1.21(0.91,1.61)	0.20
	Location (reference: rural areas)						
	Urban areas	-0.19	0.83(0.73,0.94)	<0.01	-0.19	0.83(0.76,0.90)	<0.01
	Annual per capita household income (reference: >5372 US$) [b]						
	Don’t know/not sure/refused	0.28	1.33(1.11,1.60)	<0.01	0.29	1.34(1.19,1.50)	<0.01
	< 895	0.29	1.33(1.10,1.62)	<0.01	0.29	1.33(1.18,1.51)	<0.01
	895–1789	0.12	1.13(0.91,1.40)	0.28	0.21	1.23(1.07,1.41)	<0.01
	1790–5372	0.17	1.18(0.98,1.43)	<0.01	0.17	1.19(1.06,1.33)	<0.01
	Antihypertensive medication in the last 2 weeks (reference: no)						
	Yes	-0.28	0.76(0.67,0.86)	<0.01	-0.27	0.77(0.70,0.83)	<0.01
	Number of risk factors	0.09	1.09(1.03,1.15)	<0.01	0.08	1.08(1.04,1.12)	<0.01

a: Blood pressure level: level 1 (SBP < 120 mmHg and DBP < 80 mmHg); level 2 (SBP: 120-129 mmHg and/or DBP: 80-84 mmHg); level 3 (SBP: 130-139 mmHg and/or DBP: 85-89 mmHg);level 4 (SBP: 140-159 mmHg and/or DBP: 90-99 mmHg); level 5 (SBP: 160-179 mmHg and/or DBP: 100-109 mmHg); level 6 (SBP > 180 mmHg and/or DBP > 110 mmHg)

b: Based on the exchange rate of 6.70 Chinese Yuan Renminbi per US dollar that was in effect on 30 September 2010.

Table continued.

		⩾level 6 BP [a]			⩾level 5 BP		
		Regression coefficient	OR (%95 CI)	*P*	Regression coefficient	OR (%95 CI)	*P*
Women	Education (reference: Illiterate or semi-illisteate)						
	College graduate or above	-0.77	0.46(0.30,0.73)	<0.01	-0.94	0.39(0.30,0.51)	<0.01
	Senior high school graduate or some college	-0.69	0.50(0.40,0.62)	<0.01	-0.45	0.64(0.56,0.72)	<0.01
	Junior high school graduate or some senior high school	-0.31	0.74(0.64,0.84)	<0.01	-0.27	0.76(0.70,0.83)	<0.01
	Primary school graduate or some junior high school	-0.06	0.94(0.83,1.06)	0.33	-0.08	0.92(0.85,1.00)	0.06
	Marital status (reference: single)						
	Married or cohabiting	-0.05	0.95(0.50,1.82)	0.88	0.01	1.01(0.65,1.57)	0.97
	Separated/divorced/widowed/others	-0.12	0.88(0.46,1.70)	0.71	-0.06	0.95(0.61,1.48)	0.81
	Age (reference: 18-24 years)						
	35-44 years	-0.22	0.80(0.50,1.28)	0.35	-0.03	0.98(0.71,1.34)	0.88
	45-54 years	-0.15	0.86(0.55,1.34)	0.50	-0.09	0.91(0.68,1.23)	0.55
	55-64 years	-0.30	0.74(0.48,1.15)	0.18	-0.14	0.87(0.64,1.17)	0.35
	65-74 years	0.03	1.03(0.66,1.60)	0.90	0.03	1.03(0.76,1.39)	0.87
	75+ years	0.18	1.20(0.76,1.89)	0.44	0.22	1.25(0.91,1.71)	0.17
	Location (reference: rural areas)						
	Urban areas	-0.40	0.67(0.61,0.74)	<0.01	-0.27	0.77(0.71,0.82)	<0.01
	Annual per capita household income (reference: >5372 US$) [b]						
	Don't know/not sure/refused	0.43	1.54(1.30,1.81)	<0.01	0.29	1.33(1.21,1.47)	<0.01
	< 895	0.47	1.60(1.35,1.91)	<0.01	0.31	1.37(1.23,1.53)	<0.01
	895–1789	0.48	1.62(1.35,1.96)	<0.01	0.26	1.30(1.15,1.46)	<0.01
	1790–5372	0.28	1.33(1.12,1.58)	<0.01	0.17	1.18(1.07,1.31)	<0.01
	Antihypertensive medication in the last 2 weeks (reference: no)						
	Yes	-0.07	0.94(0.84,1.05)	0.24	-0.09	0.91(0.85,0.98)	0.02
	Number of risk factors	0.11	1.12(1.05,1.18)	<0.01	0.11	1.12(1.05,1.18)	<0.01

a: Blood pressure level: level 1 (SBP < 120 mmHg and DBP < 80 mmHg); level 2 (SBP: 120-129 mmHg and/or DBP: 80-84 mmHg); level 3 (SBP: 130-139 mmHg and/or DBP: 85-89 mmHg);level 4 (SBP: 140-159 mmHg and/or DBP: 90-99 mmHg); level 5 (SBP: 160-179 mmHg and/or DBP: 100-109 mmHg); level 6 (SBP > 180 mmHg and/or DBP > 110 mmHg)

b: Based on the exchange rate of 6.70 Chinese Yuan Renminbi per US dollar that was in effect on 30 September 2010.

Table continued.

		≥level 4 BP [a]			≥level 3 BP		
		Regression coefficient	OR (%95 CI)	*P*	Regression coefficient	OR (%95 CI)	*P*
Men	Education (reference: Illiterate or semi-illisteate)						
	College graduate or above	-0.32	0.73(0.61,0.86)	<0.01	-0.17	0.85(0.68,1.06)	0.14
	Senior high school graduate or some college	-0.08	0.92(0.81,1.05)	0.22	0.03	1.03(0.86,1.23)	0.75
	Junior high school graduate or some senior high school	0.02	1.02(0.91,1.15)	0.71	0.15	1.16(0.99,1.35)	0.06
	Primary school graduate or some junior high school	0.08	1.08(0.96,1.22)	0.19	0.20	1.22(1.04,1.43)	0.01
	Marital status (reference: single)						
	Married or cohabiting	0.00	1.00(0.75,1.34)	1.00	-0.16	0.85(0.57,1.27)	0.43
	Separated/divorced/widowed/others	0.04	1.04(0.76,1.42)	0.82	-0.10	0.90(0.58,1.39)	0.64
	Age (reference: 18-24 years)						
	35-44 years	0.14	1.16(0.87,1.53)	0.31	-0.17	0.85(0.58,1.24)	0.39
	45-54 years	0.21	1.23(0.95,1.60)	0.11	-0.06	0.94(0.66,1.35)	0.75
	55-64 years	0.26	1.30(1.00,1.68)	0.05	0.01	1.01(0.71,1.44)	0.94
	65-74 years	0.31	1.36(1.04,1.77)	0.02	0.07	1.07(0.75,1.54)	0.71
	75+ years	0.44	1.55(1.17,2.06)	0.00	0.20	1.23(0.83,1.81)	0.31
	Location (reference: rural areas)						
	Urban areas	-0.14	0.87(0.80,0.95)	<0.01	-0.15	0.86(0.77,0.97)	0.01
	Annual per capita household income (reference: >5372 US$) [b]						
	Don't know/not sure/refused	0.18	1.20(1.07,1.34)	<0.01	0.11	1.11(0.96,1.30)	0.16
	< 895	0.19	1.21(1.06,1.37)	<0.01	0.04	1.04(0.88,1.23)	0.66
	895–1789	0.20	1.22(1.06,1.40)	<0.01	0.17	1.18(0.98,1.43)	0.08
	1790–5372	0.14	1.15(1.03,1.29)	0.01	0.19	1.21(1.04,1.40)	0.02
	Antihypertensive medication in the last 2 weeks (reference: no)						
	Yes	-0.19	0.83(0.76,0.91)	<0.01	-0.07	0.93(0.82,1.05)	0.23
	Number of risk factors	0.08	1.08(1.04,1.12)	<0.01	0.10	1.10(1.05,1.16)	<0.01

a: Blood pressure level: level 1 (SBP < 120 mmHg and DBP < 80 mmHg); level 2 (SBP: 120-129 mmHg and/or DBP: 80-84 mmHg); level 3 (SBP: 130-139 mmHg and/or DBP: 85-89 mmHg);level 4 (SBP: 140-159 mmHg and/or DBP: 90-99 mmHg); level 5 (SBP: 160-179 mmHg and/or DBP: 100-109 mmHg); level 6 (SBP > 180 mmHg and/or DBP > 110 mmHg)

b: Based on the exchange rate of 6.70 Chinese Yuan Renminbi per US dollar that was in effect on 30 September 2010.

Table continued.

		⩾level 4 BP [a]			⩾level 3 BP		
		Regression coefficient	OR (%95 CI)	*P*	Regression coefficient	OR (%95 CI)	*P*
Women	Education (reference: Illiterate or semi-illisteate)						
	College graduate or above	-0.74	0.48(0.40,0.58)	<0.01	-0.78	0.46(0.37,0.57)	<0.01
	Senior high school graduate or some college	-0.30	0.74(0.66,0.83)	<0.01	-0.39	0.68(0.59,0.78)	<0.01
	Junior high school graduate or some senior high school	-0.16	0.85(0.78,0.93)	<0.01	-0.16	0.85(0.75,0.96)	<0.01
	Primary school graduate or some junior high school	-0.07	0.93(0.85,1.02)	0.10	-0.09	0.92(0.81,1.03)	0.15
	Marital status (reference: single)						
	Married or cohabiting	0.01	1.01(0.64,1.57)	0.98	-0.21	0.81(0.43,1.50)	0.50
	Separated/divorced/widowed/others	-0.01	0.99(0.63,1.55)	0.95	-0.25	0.78(0.41,1.45)	0.43
	Age (reference: 18-24 years)						
	35-44 years	-0.17	0.85(0.62,1.16)	0.30	-0.23	0.79(0.54,1.16)	0.24
	45-54 years	-0.03	0.97(0.72,1.31)	0.85	0.04	1.04(0.73,1.50)	0.82
	55-64 years	-0.03	0.97(0.72,1.30)	0.83	0.11	1.12(0.78,1.60)	0.55
	65-74 years	0.11	1.12(0.83,1.51)	0.46	0.16	1.18(0.82,1.70)	0.38
	75+ years	0.18	1.20(0.87,1.64)	0.26	0.12	1.13(0.77,1.65)	0.55
	Location (reference: rural areas)						
	Urban areas	-0.25	0.78(0.72,0.83)	<0.01	-0.22	0.80(0.73,0.88)	<0.01
	Annual per capita household income (reference: >5372 US$) [b]						
	Don’t know/not sure/refused	0.20	1.22(1.10,1.34)	<0.01	0.23	1.26(1.11,1.43)	<0.01
	< 895	0.21	1.23(1.10,1.37)	<0.01	0.13	1.14(0.99,1.32)	0.06
	895–1789	0.26	1.30(1.15,1.47)	<0.01	0.22	1.25(1.07,1.46)	<0.01
	1790–5372	0.16	1.17(1.06,1.29)	<0.01	0.12	1.13(0.99,1.28)	0.07
	Antihypertensive medication in the last 2 weeks (reference: no)						
	Yes	-0.02	0.98(0.90,1.06)	0.59	0.08	1.08(0.97,1.20)	0.14
	Number of risk factors	0.13	1.14(1.10,1.19)	<0.01	0.16	1.20(1.14,1.27)	<0.01

a: Blood pressure level: level 1 (SBP < 120 mmHg and DBP < 80 mmHg); level 2 (SBP: 120-129 mmHg and/or DBP: 80-84 mmHg); level 3 (SBP: 130-139 mmHg and/or DBP: 85-89 mmHg);level 4 (SBP: 140-159 mmHg and/or DBP: 90-99 mmHg); level 5 (SBP: 160-179 mmHg and/or DBP: 100-109 mmHg); level 6 (SBP > 180 mmHg and/or DBP > 110 mmHg)

b: Based on the exchange rate of 6.70 Chinese Yuan Renminbi per US dollar that was in effect on 30 September 2010.

Table continued

		⩾level 2 BP [a]		
		Regression coefficient	OR (%95 CI)	*P*
Men	Education (reference: Illiterate or semi-illisteate)			
	College graduate or above	0.07	1.07(0.74,1.55)	0.72
	Senior high school graduate or some college	0.14	1.15(0.86,1.53)	0.35
	Junior high school graduate or some senior high school	0.23	1.26(0.99,1.61)	0.06
	Primary school graduate or some junior high school	0.39	1.48(1.14,1.91)	<0.01
	Marital status (reference: single)			
	Married or cohabiting	-0.11	0.90(0.46,1.76)	0.76
	Separated/divorced/widowed/others	-0.13	0.88(0.43,1.79)	0.71
	Age (reference: 18-24 years)			
	35-44 years	0.26	1.30(0.71,2.38)	0.39
	45-54 years	0.11	1.11(0.64,1.93)	0.70
	55-64 years	0.04	1.05(0.61,1.80)	0.87
	65-74 years	-0.02	0.98(0.56,1.69)	0.93
	75+ years	0.06	1.06(0.58,1.92)	0.85
	Location (reference: rural areas)			
	Urban areas	-0.20	0.82(0.67,0.99)	0.04
	Annual per capita household income (reference: >5372 US$) [b]			
	Don’t know/not sure/refused	0.03	1.03(0.80,1.34)	0.80
	< 895	-0.29	0.75(0.57,0.98)	0.04
	895–1789	0.16	1.18(0.85,1.63)	0.33
	1790–5372	0.02	1.02(0.79,1.32)	0.87
	Antihypertensive medication in the last 2 weeks (reference: no)			
	Yes	0.16	1.17(0.97,1.42)	0.10
	Number of risk factors	0.12	1.13(1.04,1.23)	<0.01

a: Blood pressure level: level 1 (SBP < 120 mmHg and DBP < 80 mmHg); level 2 (SBP: 120-129 mmHg and/or DBP: 80-84 mmHg); level 3 (SBP: 130-139 mmHg and/or DBP: 85-89 mmHg);level 4 (SBP: 140-159 mmHg and/or DBP: 90-99 mmHg); level 5 (SBP: 160-179 mmHg and/or DBP: 100-109 mmHg); level 6 (SBP > 180 mmHg and/or DBP > 110 mmHg)

b: Based on the exchange rate of 6.70 Chinese Yuan Renminbi per US dollar that was in effect on 30 September 2010.

Table continued

		⩾level 2 BP [a]		
		Regression coefficient	OR (%95 CI)	*P*
Women	Education (reference: Illiterate or semi-illisteate)			
	College graduate or above	-0.55	0.58(0.41,0.81)	<0.01
	Senior high school graduate or some college	-0.30	0.74(0.59,0.94)	<0.01
	Junior high school graduate or some senior high school	-0.13	0.88(0.73,1.07)	0.20
	Primary school graduate or some junior high school	-0.07	0.94(0.77,1.13)	0.50
	Marital status (reference: single)			
	Married or cohabiting	-0.12	0.89(0.31,2.51)	<0.01
	Separated/divorced/widowed/others	-0.12	0.89(0.31,2.55)	<0.01
	Age (reference: 18-24 years)			
	35-44 years	-0.20	0.81(0.49,1.34)	0.42
	45-54 years	0.31	1.36(0.85,2.19)	0.20
	55-64 years	0.48	1.61(1.00,2.59)	0.05
	65-74 years	0.37	1.45(0.89,2.36)	0.14
	75+ years	0.44	1.55(0.91,2.62)	0.10
	Location (reference: rural areas)			
	Urban areas	-0.22	0.80(0.68,0.94)	0.01
	Annual per capita household income (reference: >5372 US$) [b]			
	Don't know/not sure/refused	0.26	1.29(1.06,1.58)	0.01
	< 895	0.11	1.12(0.89,1.40)	0.33
	895–1789	0.29	1.34(1.04,1.73)	0.02
	1790–5372	0.16	1.17(0.96,1.43)	0.13
	Antihypertensive medication in the last 2 weeks (reference: no)			
	Yes	0.31	1.37(1.16,1.60)	<0.01
	Number of risk factors	0.16	1.17(1.08,1.28)	<0.01

a: Blood pressure level: level 1 (SBP < 120 mmHg and DBP < 80 mmHg); level 2 (SBP: 120-129 mmHg and/or DBP: 80-84 mmHg); level 3 (SBP: 130-139 mmHg and/or DBP: 85-89 mmHg);level 4 (SBP: 140-159 mmHg and/or DBP: 90-99 mmHg); level 5 (SBP: 160-179 mmHg and/or DBP: 100-109 mmHg); level 6 (SBP > 180 mmHg and/or DBP > 110 mmHg)

b: Based on the exchange rate of 6.70 Chinese Yuan Renminbi per US dollar that was in effect on 30 September 2010.
